# Children with Hirschsprung's Disease and Syndromes with Cognitive Dysfunction: Manifestations, Treatment, and Outcomes

**DOI:** 10.1055/s-0039-1696730

**Published:** 2019-09-04

**Authors:** Josefine Hedbys, Johan Hasserius, Christina Granéli, Einar Arnbjörnsson, Kristine Hagelsteen, Pernilla Stenström

**Affiliations:** 1 Department of Clinical Sciences, Lund University, Lund, Sweden; 2 Department of Pediatric Surgery, Skåne University Hospital, Lund, Sweden

**Keywords:** Hirschsprung's disease, pediatrics, bowel function, cognitive dysfunction, transanal endorectal pull-through, long-term outcome

## Abstract

**Introduction** To assess differences in initial symptoms, treatments, and bowel function between children with Hirschsprung's disease (HD) with or without a cognitive dysfunction (CD).

**Materials and Methods** The study included children with HD who underwent transanal endorectal pull-through. A retrospective chart review was performed to collect data on patient characteristics, diagnosis, and treatment. Data on bowel symptoms in children older than 4 years without a colostomy were compiled during a cross-sectional, patient-reported follow-up.

**Results** Fifty-three children with HD were included; of these, 12 (23%) had CD. The median birth weight was lower, frequency of vomiting as the presenting symptom was lower, and time until the first contact with a pediatric surgeon was higher in children with CD than in those without (3,295 vs. 3,623 g, *p* = 0.013; 28 vs. 66%, *p* = 0.02; and 4 days vs. 1 day, *p* = 0.048, respectively). At follow-up, 5 (15%) of 33 children aged over 4 years had CD. More children without CD had some ability to hold back defecation and sense the urge to defecate than those with CD ( *p* = 0.002 and *p* = 0.001, respectively).

**Conclusion** HD children who have CD present with different initial symptoms, have a delay in the first consultation with a pediatric surgeon, and experience poorer bowel function outcomes than HD children without CD. Therefore, HD children with CD should receive special attention in both clinical practice and research.

Hirschsprung's disease (HD) is a rare congenital bowel disorder, with an incidence of 1 per 5,000 births, and 10 to 15% of HD patients have been reported to also have trisomy 21. 1 2 3 4 5 Bowel control is one of the most important outcomes in children with HD, and it depends upon the patient's overall functional and cognitive ability, the patient's own motivation, and parental support. 6 7 Considering these findings, cognitive dysfunction (CD) in children might have a negative influence on bowel control. This would explain why children with CD are often excluded from long-term follow-up. 8 Children with HD and CD might receive different treatment than children with only HD. Some researchers have suggested that all patients with HD and trisomy 21 should undergo a colostomy due to their poor prognosis, 4 6 whereas others have recommended that children with CD should receive the same treatment as that used in children without. 5 9 10 Information on the short- and long-term outcomes in children with HD and CD is limited, and reports on the pre- and postoperative care of these patients are lacking.

The aim of this study was to assess differences in patient features, initial symptoms, diagnostics, preoperative and initial postoperative treatments, and long-term bowel function outcomes between children with HD and CD and those with only HD treated with transanal endorectal pull-through (TERPT). Our findings might help improve the care and treatment of children with HD.

## Materials and Methods

### Settings

This study was conducted at a tertiary pediatric surgery center that covers 2 million residents and 25,000 live births per year. The study had a dual design. One part of the study involved retrospective evaluation of the records of children with HD, and another part involved cross-sectional, long-term follow-up comparing bowel symptoms between HD children with CD and those without CD.

For the retrospective analysis, patient data including birth characteristics, initial symptoms, preoperative data, and perioperative care data were collected from medical charts. Data on postoperative status, including the number of counseling sessions during the first year after surgery and frequencies of anorectal complications, calibrations, and anastomosis dilatations, were compiled within 1 year postoperatively. Two independent researchers (JHa and JHe) who had no previous association with the patients collected and compared data to ensure interrater reliability.

The long-term follow-up was performed during 2016 in the outpatient clinic at the annual follow-up counseling session. Bowel function was assessed using the Rintala bowel function score (BFS), which has been evaluated and used in several previous HD studies. 11 12 The total score ranges from 1 to 20 points, where a score of 18 or higher indicates normal bowel function and a score below 15 indicates severely impaired bowel function. 13 If children were able to understand and answer the questions, they completed the questionnaire together with their parents; otherwise, the legal guardian answered the questionnaire alone.

### Patients

The study included children born between July 2006 and December 2015. All children diagnosed with HD and treated with TERPT at our department were included. We excluded children who were treated with other methods, those treated at other departments, those who had emigrated, and those who had total colonic aganglionosis. To evaluate bowel function, children younger than 4 years and those with a colostomy were excluded.

### Diagnosis and Surgical Procedure

HD was diagnosed with suction or open rectal biopsy. To evaluate the position of the transition zone prior to the pull-through, cold contrast enema was performed. 14 TERPT was initiated by transanal mucosectomy starting approximately 10 mm above the dentate line and reaching another 2 to 4 cm, leaving a muscle cuff. TERPT was performed either entirely transanal or with laparoscopic-assisted colonic mobilization, assuming the left flexure of the colon required mobilization. Mobilization of the colon was performed to a region above the transition zone. Frozen-section biopsy-confirmed ganglion cells in the bowel were pulled down to the rectum before anastomosis was established. Some children had received a colostomy preoperatively; in those cases, stoma closure was performed at the same time as pull-through. After TERPT, all patients were treated according to a postoperative program that included regular counseling and anal calibration. All surgeries and the postoperative follow-up were performed by the same three pediatric surgeons who had colorectal profiles.

### Criteria for Cognitive Dysfunction

CD was defined as significant cognitive ability falling in the borderline/low range reported by children's parents. A key indicator for a cognitive disability was a lack of self-help skills or lack of ability to perform daily tasks such as showering, getting dressed, learning at school, and so on, without support. 15

### Definitions

Delayed meconium release was defined as not passing meconium without assistance within 48 hours postpartum. Calibration was defined as anastomosis measurement using a Hegar dilator, Foley catheter, or measured finger. Dilatation was defined as a procedure intended to widen the anastomosis using a Hegar dilator with the patient under sedation or anesthesia. Stricture was defined as narrowing of the anastomosis, requiring dilatation. According to the local bowel management program, the indications for appendicostomy were need for regular colonic washout, need for greater autonomy, and unwillingness or refusal to undergo rectal enema due to intolerance to rectal manipulation.

### Statistical Analysis

Nonparametric statistical tests were applied because of the small sample size. Fisher's two-tailed exact test was used for dichotomous results. Continuous quantitative variables and tri- and tetrachotomous results were analyzed using the Mann–Whitney *U* test. All statistical analyses were performed using SPSS Statistics 23 (IBM Corp., Armonk, NY). A *p* -value of <0.05 was considered statistically significant. A statistician designed and supported the statistical calculations in the study.

### Ethical Considerations

All treatments and procedures were performed according to the standard care protocol for children with HD at the center for pediatric surgery. All patient data from medical charts and answers to questionnaires or interviews were collected in a database on a continuous basis. To ensure anonymity, all children were assigned encoded numbers prior to calculations. Each patient/parent received verbal and written information about the study beforehand. Each patient's guardian provided written consent to participate in this study. The study was approved by the Regional Ethical Review Board (registration number 2010/49). The included children were registered after obtaining consent, according to regional demands, in a quality register (number 01481271007173). Administrative permission for access to medical records was received from the hospital.

## Results

### Patients

**Fig. 1:**
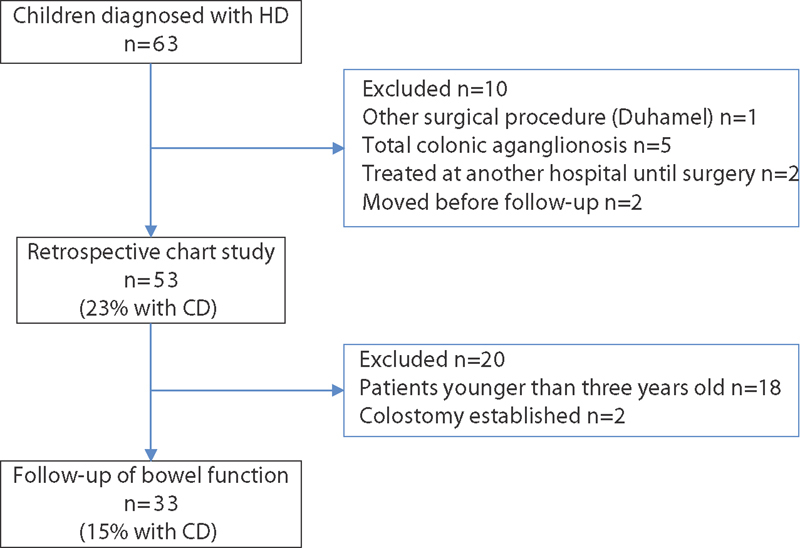
Overview of patients with HD who underwent surgery from July 2006 until December 2015 and included in this study. CD, cognitive dysfunction; HD, Hirschsprung's disease.

A total of 63 children were diagnosed with HD and underwent surgery during the study period. Of these, 53 children were included in the retrospective chart analysis ( Fig. 1 ). Among these 53 children, 12 (23%) met the criteria for CD ( Table 1 ).

**Table 1:** Overview of children with CD present among the 53 patients with Hirschsprung's disease

Type of CD	*N* (%)
Trisomy 21	7 (13)
BRESCHEK a	1 (2)
Other chromosomal abnormalities	1 (2)
Strong suspicion of a syndrome, ongoing analysis	3 (6)
Total	12 (23)

Abbreviation: CD, cognitive dysfunction.

a *BRESHECK syndrome* is caused by a mutation in *MBTPS2* and includes a multiple congenital malformation as summarized by the acronym 16 : *B* rain anomalies, *R* etardation of mentality and growth, *E* ctodermal dysplasia, *S* keletal defects, *H* irschsprung's disease, *E* ar deformity and deafness/eye hypoplasia, *C* left palate/ *C* ryptorchidism, *K* idney dysplasia/hypoplasia.

In the follow-up for bowel function, among the 53 children, 20 were excluded (18 had not yet reached 4 years of age and 2 had a colostomy). Thus, bowel symptoms were eventually assessed in 33 children, including 5 (15%) children with a syndrome and 28 (85%) children without a syndrome ( Fig. 1 ). At follow-up, the children's median age, both with and without CD, was 7 years (range: 4–10). In the retrospective analysis, 10 (24%) of the children without CD and 2 (17%) with CD were girls. In the follow-up, 8 (29%) of the children without CD and 1 (20%) of the children with CD were girls.

### Patient Characteristics and Initial Symptoms

The median birth weight was significantly lower in children with CD than in those without (3,295 g [range: 2,100–3,890 g] vs. 3,623 g [range: 2,355–4,675 g], respectively; *p* = 0.013). On the other hand, the median gestation age did not differ between children with CD and those without CD (38.5 weeks [range: 36–42 weeks] and 39.5 weeks [range: 32–42 weeks], respectively; *p* = 0.279).

**Fig. 2:**
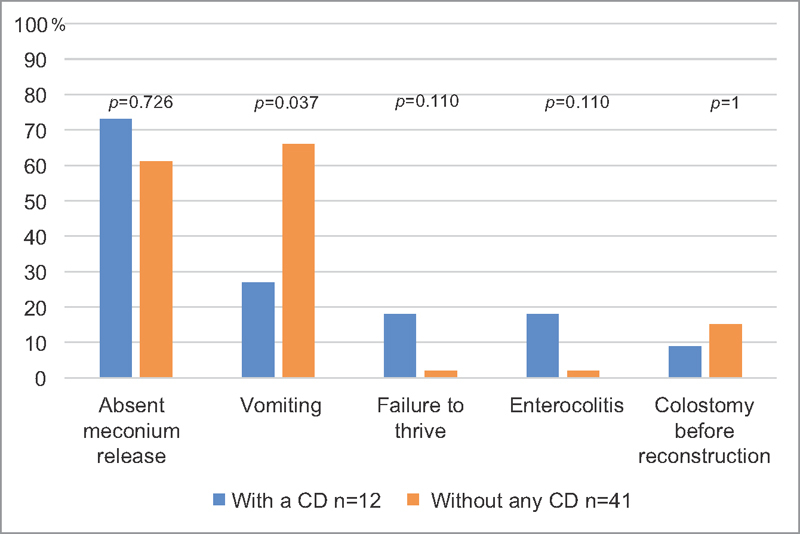
Presenting symptoms and colostomy before transanal endorectal pull-through for Hirschsprung's disease in children with and without CD. CD, cognitive dysfunction.

Vomiting was the most common symptom in children without CD, and it was significantly more common in children without CD. The absence of meconium release was the most common initial symptom in children with CD; however, the frequency did not differ between children with CD and those without CD. Moreover, failure to thrive tended to be more common in children with CD than in those without CD ( Fig. 2 ).

The median age at symptom onset was 1 day in children with and without CD. Contact with a pediatric surgeon after initial symptoms was later in children with CD than in those without CD (4 days vs. 1 day). There were no significant differences in the time for diagnostic procedures, time from diagnosis to surgery, and age at TERPT between children with CD and those without CD ( Table 2 ).

**Table 2:** Diagnostic preoperative care in 53 patients with HD with and without a CD

	HD patients with CD		HD patients without any CD		*p* -Value a
	*n*	Days, median (range)	*N*	Days, median (range)	
Age at symptom onset	11	1 (1–167)	41	1 (1–228)	0.669
Age at the first contact with a pediatric surgeon	12	7 (1–1,119)	41	2 (1–1,054)	0.066
Age at rectal biopsy	12	11 (5–1,138)	41	7 (1–1,054)	0.120
Age at diagnosis	12	23 (9–1,159)	40 1	24 (7–1,072)	0.633
Age at TERPT	12	50 (12–1,279)	41	50 (15–1,254)	0.848
Time between the first symptom and contact with a pediatric surgeon	11	4 (0–952)	41	1 (0–952)	0.048
Time between biopsy result and operation	12	4.5 (0–90)	39	5 (−2–367)	0.592
Time between biopsy result and reconstruction	12	22 (2–120)	40	27.5 (1–418)	0.587
Time between the first contact with a pediatric surgeon and reconstruction	12	36 (9–160)	41	38 (12–888)	0.655

Abbreviations: CD, cognitive dysfunction; HD, Hirschsprung's disease; TERPT, transanal endorectal pull-through.

a Mann–Whitney *U* test, two-tailed.

### Postoperative Results

The median hospital stays after TERPT tended to be longer in children with CD (5 vs. 4 days). There was no significant difference in the number of planned or emergency outpatient visits or the number of hospitals stays within the first year postoperatively. There were no differences in postoperative complications such as anal skin excoriation, anastomotic strictures, and need for anal dilatations during the first postoperative year ( Table 3 ). In the interval between TERPT and the long-term follow-up, 2 (17%) of the children with CD and 3 (7%) of the children without CD received a colostomy ( *p* = 0.315).

**Table 3:** Postoperative care and symptoms during the first year after TERPT in 53 children with HD with and without CD

	Children with CD ( *n* = 12)	Children without CD ( *n* = 41)	*p* -value
Postoperative hospital stay (days)	5 (3–22)	4 (1–13)	0.036 a
Time between leaving the hospital and the first counseling postoperatively (days)	9 (2–23)	11 (2–39)	0.418 a
HD consultations 1 y postoperatively ( *n* )	11 (1–23)	8 (2–38)	0.903 a
Acute HD consultations 1 y postoperatively ( *n* )	1 (0–5)	0 (0–11)	0.193 a
Hospital stay owing to HD 1 y postoperatively (n)	1 (0–4)	0 (0–6)	0.634 a
Anal calibrations 1 y postoperatively ( *n* )	7 (1–23)	6 (1–26)	0.674 a
Anal dilatations 1 y postoperatively ( *n* )	0 (0–4)	0 (0–9)	0.698 a
Perianal skin excoriations	7 (58)	14(34)	0.183 b
Anastomotic stricture	1 (8)	9 (22)	0.423 b

Abbreviations: CD, cognitive dysfunction; HD, Hirschsprung's disease; TERPT, transanal endorectal pull-through.

Note: data are presented as median (range) or number (%).

a Mann–Whitney *U* test, two-tailed.

b Fisher's exact test, two-tailed.

### Bowel Function

In the long-term follow-up, two children had stomas and were, therefore, excluded (one child with CD had a severe anastomotic stricture and one child without CD had a urinary–rectal fistula). At the time of follow-up, appendicostomy was performed in two (40%) of the children with CD and two (11%) without CD ( *p* = 0.155). These patients were included as enema users.

All children with CD reported at least weekly problems holding back defecation, whereas the responses for children without CD were more dispersed, ranging from “always able to hold back defecation” to “weekly problems” ( Table 4 ). All children without CD reported voluntary bowel control, whereas significantly more children with CD (60%) reported an absence of control. Most children with CD (60%) reported that they did not feel the urge to defecate, whereas most without CD (46%) reported that they felt the urge to defecate on most occasions ( Table 4 ).

**Table 4:** Bowel function in 33 children with Hirschsprung's disease with and without CD

Bowel symptoms				*p* -Value a
		With CD	Without CD	
		*n* = 5	*n* = 28	
Ability to hold back defecation				*0.002*
Always	3	0	5 (18)	
Problems < one time per week	2	0	11 (39)	
Weekly problems	1	2 (40)	12 (43)	
No voluntary control	0	3(60)	0	
Feeling of the urge to defecate				*0.001*
Always	3	0	8 (29)	
Most of the time	2	0	13 (46)	
Uncertain	1	2 (40)	7 (25)	
Absent	0	3 (60)	0	
Frequency of defecation				0.173
Every other day to twice a day	2	1 (25)	15 (54)	
More often	1	4 (75)	13 (46)	
Less often	0	0	0	
Soiling				0.093
Never	3	1 (20)	0	
Staining < one time\ per /week, no change of underwear required	2	0	14 (50)	
Frequent staining, change of underwear often required	1	1 (20)	12 (43)	
Daily soiling, requires protective aids	0	3 (60)	2 (7)	
Fecal accidents				0.217
Never	3	2 (40)	13 (46)	
Fewer than one time per week	2	0	10 (36)	
Weekly, requires protective aids	1	1 (20)	4 (14)	
Daily, requires protective aids day and night	0	2 (40)	1 (4)	
Constipation				0.095
No constipation	3	2 (40)	19 (68)	
Manageable with diet	2	0	6 (21)	
Manageable with laxatives	1	2 (40)	2 (7)	
Manageable with enema	0	1 (20)	1 (7)	

Abbreviations: CD, cognitive dysfunction.

Note: data are presented as number (%).

a Mann–Whitney *U* test.

The total BFS for children with CD could not be evaluated, as they were not able to answer the question about social functioning. In the 28 children without CD, the median total BFS was 14 (range: 8–20). Among these children, 3 (11%) had scores indicating normal bowel function (18–20) and 16 (57%) had scores indicating a poor outcome (<15).

## Discussion

In this study, we found that HD children with CD had a lower birth weight, a longer time from initial symptoms to the first contact with a pediatric surgeon, a longer hospitalization after reconstruction, and different initial HD symptoms when compared with the findings in HD children without CD. Additionally, in long-term follow-up, bowel function was less favorable in children with CD than in those without CD. Thus, our study provides novel information on HD children with CD.

Limited previous reports are available in the literature on children with HD and syndromes, and all focus on trisomy 21. 17 18 19 20 The proportion of patients with HD and trisomy 21 in our study (13%) is similar to that in previous studies (13–15%). 17 18 19 However, as noted in Table 5 , our study differed from previous studies in that children with syndromes with CD were included, only children were included (not adults), and all patients underwent TERPT instead of various other surgical techniques.

**Table 5:** Data from previous studies on children with Hirschsprung's disease and trisomy 21

Author 17 18 19 20	Year	Patients with trisomy 21, *n* (% of total)	Boys:girls	Method of surgery	Stoma %	Constipation %	laxatives %	Enema a %	Diarrhea %	Follow-up (years)
Hackam et al	2003	9 (14)	8:1	Soave b	67	33	–	–	11	1.8
Menezes and Puri	2005	39 (15)	29:10	Soave b	59	30.4 c		30.4 c	–	0.5–28
Catto-Smith et al	2006	21 (5)	9:1	Soave b	–	60	50	10	40	3.4–17.2
Travassos et al	2011	20 (13)	14:6	Duhamel	–	75 d	20	–	–	5.1

a Treatment on a regular basis.

b Different methods of surgery were used; the most common method is stated.

c 30.4% of the patients received either laxatives or enemas regularly.

d 75% had constipation and 55% were considered to be severely constipated.

Identifying HD through the recognition of initial symptoms is important to avoid any delay in HD diagnosis and reduce the risk of developing fulminant enterocolitis. Although children with CD in our study had classical initial symptoms of HD, 19 their overall presentation differed somewhat from that of children without CD. This difference might be associated with the delayed contact with a pediatric surgeon identified among children with CD. Previous studies have not reported a difference in symptoms or a delay in contact with a pediatric surgeon among HD children with CD. Thus, our findings need attention in further studies, and in the clinical setting, to provide equal medical care to all HD children. It is important to note that hospital stay after TERPT was longer in children with CD than in those without CD. A previous study reported a longer hospital stay after surgery for HD among children with trisomy 21 than those without (9.5 vs. 7 days), and this increased hospital stay was associated with the management and treatment of complications related to various additional comorbidities. 18 The general status of the child at the time of diagnosis could play a role in the postoperative length of hospital stay. If the loss of time before contact with a pediatric surgeon can be avoided among children with any CD, the starting point for treatment might improve, resulting in a shorter hospital stay. A longer hospital stay might also be associated with the time before complete enteral feeding. However, the time before accomplishing complete enteral feeding was not assessed in this study; to our knowledge, this factor has not been reported in any previous study on HD patients with CD but could be of interest in future studies.

The ability to hold back defecation and sense the urge to defecate differed significantly between children with and without CD. These findings are consistent with previous findings in children with trisomy 21. 17 18 20 21 However, to our knowledge, this study is the first to evaluate bowel function in HD children with CD as a common factor. Some studies have suggested that a poor bowel function outcome might be associated with the child's social or cognitive abilities and intelligence, which might be related to poor motivation and ability to achieve normal toilet training. 17 21 The reasons for reduced bowel function should be assessed since they could be of importance in helping children with CD achieve better bowel function and quality of life.

This study has some strengths worth mentioning. First, by choosing to include all children with CD, we could report on a group of children not previously mentioned or studied in the literature. Second, only children who underwent TERPT were included, and the same surgeons performed all operations, ensuring comparable results. Third, the chart system was almost entirely absolute as all documentation followed a standard template of diagnosis and follow-up, enabling thorough and trustworthy data collection, although the study was retrospective.

The study had several limitations. A small number of children were included, and the number of patients with CD was even lower. Therefore, it is difficult to draw valid conclusions from our data. This might be the reason why some significant differences were not noted, although strong trends were identified (fecal accidents, soiling, and constipation). This strongly indicates a statistical type 2 error. Larger studies are needed to draw accurate conclusions, and this study could be a good platform to calculate power for such future studies. Limitations were also present because of the retrospective chart study design, and some interpretations of answers had to be made, which may have influenced the results. A prospective study would overcome these limitations.

Children with CD represent a large portion of those afflicted with HD, and adapting the BFS and grading system for cognitive functionality could provide more accurate results in future studies. No clear guidelines exist on which children should be considered as having neurologic syndromes or CD. Children with CD (especially those with trisomy 21) are often excluded from follow-up. As questions about social problems are often difficult to answer for children with CD, we fear that this might contribute to the exclusion of this patient group. Therefore, we suggest a modified scoring system for bowel function, with adapted scores for social problems in children with CD. Many children with CD need special care both before and after surgery for HD. The study results suggest close cooperation between pediatricians and pediatric surgeons, with the aim of a better outcome in this group of children.

## Conclusion

HD children who later will develop CD present with different initial symptoms, have a delay in the first consultation with a pediatric surgeon, and experience poorer bowel function outcomes than HD children without CD. Therefore, HD children with CD should receive special attention in both clinical practice and research.
